# The crystal structure of ((cyclo­hexyl­amino){(*Z*)-2-[(*E*)-5-meth­oxy-3-nitro-2-oxido­benzyl­idene-κ*O*]hydrazin-1-yl­idene-κ*N* ^2^}methane­thiol­ato-κ*S*)(dimethyl sulfoxide-κ*S*)platinum(II): a supra­molecular two-dimensional network

**DOI:** 10.1107/S2056989019012623

**Published:** 2019-09-12

**Authors:** Md. Azharul Arafath, Huey Chong Kwong, Farook Adam

**Affiliations:** a Department of Chemistry, Shahjalal University of Science and Technology, Sylhet 3114, Bangladesh; b School of Chemical Sciences, Universiti Sains Malaysia, Penang 11800 USM, Malaysia

**Keywords:** crystal structure, cyclo­hexyl­hydrazinecarbo­thio­amide, soft Lewis base, carbo­thio­amide Schiff base, hydrogen bonding, C—H⋯π inter­actions

## Abstract

The Pt^II^ atom in the title complex, [Pt(C_15_H_18_N_4_O_4_S)(C_2_H_6_OS)], exists within a square-planar NS_2_O donor set provided by the N, S, O atoms of the di-anionic tridentate thio­semicarbazo ligand and a dimethyl sulfoxide S atom. The two chelate rings are coplanar, subtending a dihedral angle of 1.51 (7)°. The maximum deviation from an ideal square-planar geometry is seen in the five-membered chelate ring with an S—Pt—S bite angle of 96.45 (2)°. In the crystal, mol­ecules are linked *via* N—H⋯O, C—H⋯O, C—H⋯N and C—H⋯π inter­actions into two-dimensional networks lying parallel to the *ab* plane. The conformations of related cyclo­hexyl­hydrazine-1-carbo­thio­amide ligands are compared to that of the title compound.

## Abstract 2

The title complex consists of a Pt^II^ atom coordinated in a square-planar environment by a dimethyl sulfoxide mol­ecule and a thio­semicarbazone ligand. The overall conformation of the title complex is discussed and compared with related ligands. In the crystal, mol­ecules are assembled *via* hydrogen bonds and C–H⋯π inter­actions forming a two-dimensional network.

## Chemical context   

Schiff base ligands and their complexes with transitional metals form an important functionality in medicinal, industrial and coordination chemistry (Hanifehpour *et al.*, 2015 ▸; Singh *et al.*, 2007 ▸). Cisplatin was synthesized by Peyrone in 1844 (Peyrone, 1844 ▸), and its use for treatment against human cancer was authorized in 1978, the biological effects of this compound on cancer cells having been discovered serendipitously by Rosenberg and co-workers in 1965 (Rosenburg *et al.*, 1965 ▸). Work by medicinal chemists on the coordination and biological properties of metal complexes has contributed to the emergence of modern medicinal chemistry, which was inspired by the discovery of cisplatin. The thio­semicarbazone moiety containing Schiff base ligands chelated to platinum(II) shows high anti­tumor and anti­cancer activity; metal-based drugs are more promising and convenient as therapeutic agents (Nomiya *et al.*, 1998 ▸; Kovala-Demertzi *et al.*, 2003 ▸; Kovala-Demertzi *et al.*, 2000 ▸; Anacona *et al.*, 1999 ▸; Arafath *et al.*, 2017*b* ▸). The complexes of Pt^II^ with 4(*N*)-substituted deriv­atives of 2-acetyl­pyridine thio­semicarbazone exhibit potential anti­tumor, anti­cancer, anti­bacterial, anti­neoplastic and cytogenetic activities (Kovala-Demertzi *et al.*, 1999 ▸, 2000 ▸, 2001 ▸). Carbazate containing N- and S-coordinating sites chelated to Pt^II^ exhibits potential anti­bacterial and anti­cancer activity (Tarafder *et al.*, 2002 ▸; Arafath *et al.*, 2019 ▸). Herein we describe the synthesis and crystal structure of one such complex, ((cyclo­hexyl­amino){(*Z*)-2-[(*E*)-5-meth­oxy-3-nitro-2-oxido­benzyl­idene-κ*O*]hydrazin-1-yl­idene-κ*N* ^2^}methane­thiol­ato-κ*S*)(dimethyl sulfoxide-κ*S*)platinum(II), **I**.
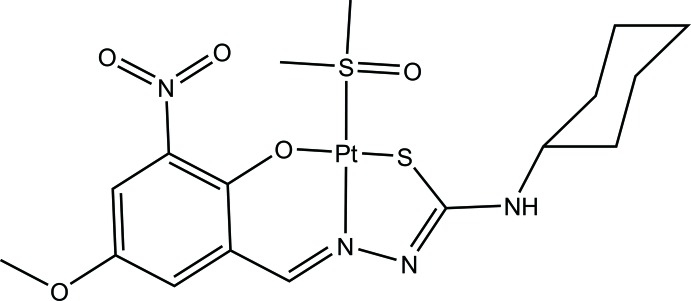


## Structural commentary   

**Figure 1:**
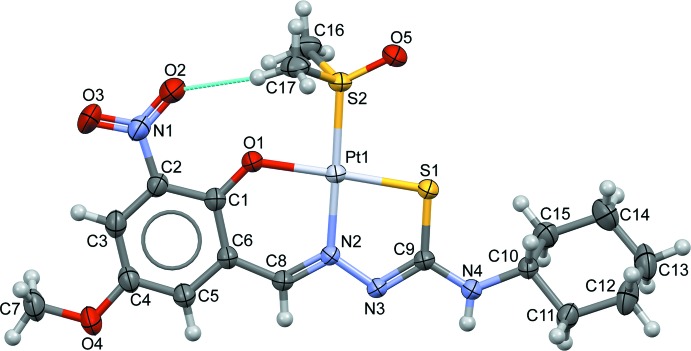
The mol­ecular structure of complex **I**, with atom labelling and displacement ellipsoids drawn at the 50% probability level.

The mol­ecular structure of complex **I** is shown in Fig. 1 ▸. Selected geometrical parameters involving atom Pt1 are given in Table 1 ▸. The Pt^II^ atom is four-coordinate, creating a square-planar PtNOS_2_ environment with a maximum deviation of 0.0105 (2) Å for atom N2. The coordination environment consists of a thio­semicarbazone and a dimethyl sulfoxide mol­ecule. The thio­semicarbazone mol­ecule coordinates in a tridentate manner through thio­amide sulfur atom S1, azo­meth­ine nitro­gen N2 and phenyl oxygen O1, creating two chelate rings which are coplanar; the dihedral angle between the mean planes of the five-membered Pt1/S1/N2/N3/C9 and six-membered Pt1/O1/N2/C1/C6/C8 chelate rings is 1.51 (7)°. The benzene ring (C1–C6) is almost coplanar with both chelate rings, making dihedral angles of 2.82 (9) and 1.36 (10)°, respectively. The bite angles formed between the thio­semicarbazone ligand and the metal are N2—Pt1—S1 = 84.74 (5)° and O1—Pt1—N2 = 93.95 (6)°. The angles formed between the thio­semicarbazone, metal and the dimethyl sulfoxide are O1—Pt1—S2 = 84.87 (5)° and S1—Pt1—S2 = 96.45 (2)°. As a result of chelation, the two azomethine C=N double bonds, N2=C8 and N3=C9, are in *Z* and *E* configurations, respectively. This leads to both azomethine double bonds adopting a *s-trans* conformation with respect to each other. The cyclo­hexane ring adopts a chair conformation with puckering amplitude *Q* = 0.567 (3) Å, θ = 175.9 (3)° and φ = 160 (5)°, and forms a torsion angle (C9—N4—C10—C11) of 162.2 (2)° to the Pt1/S1/N2/N3/C9 chelate ring. In the 5-meth­oxy-3-nitro-2-oxidobenzyl ring, the meth­oxy and nitro groups are almost coplanar with the benzene ring, as indicated by the torsion angles O2—N1—C2—C1 = 4.0 (4)° and C7—O4—C4—C3 = 4.7 (4)°. Oxygen atom O5 of the dimethyl sulfoxide mol­ecule is almost coplanar with both chelate rings [O5—S2—Pt1—S1 = 1.83 (11)°], whereas the methyl groups are twisted with respect to the chelate ring [C16—S2—Pt1—S1 =123.64 (11) and C17—S2—Pt1—S1 = −127.60 (12) °]. In the mol­ecule, atom O2 of the nitro group acts as a hydrogen-bond acceptor for the adjacent methyl group, forming an intra­molecular C—H⋯O hydrogen bond with an *S*(9) ring motif (Fig. 1 ▸, Table 2 ▸).

## Supra­molecular features   

**Figure 2:**
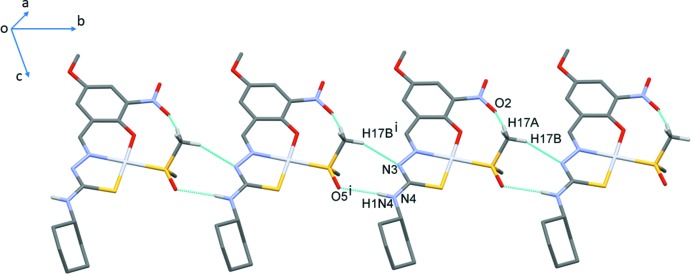
A view of the hydrogen-bonded chain formed by N—H⋯O and C—H⋯N hydrogen bonds [dashed lines; symmetry code: (i) *x*, *y* − 1, *z*]. Hydrogen atoms not involved in these inter­actions have been omitted for clarity.

**Figure 3:**
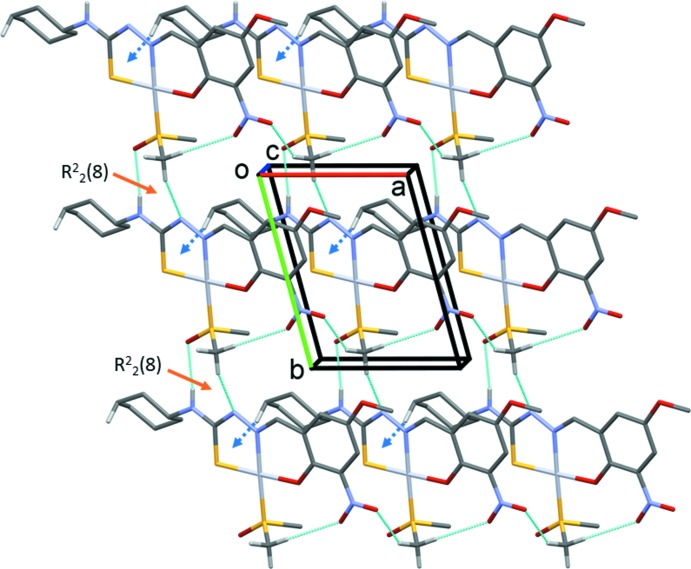
A view along the *c* axis of the crystal packing of complex **I**. The hydrogen bonds are shown as dashed lines and the C—H⋯π inter­actions are represented as dashed blue arrows (Table 1 ▸). Hydrogen atoms not involved in these inter­actions have been omitted for clarity.

In the crystal of **I**, mol­ecules are linked by N4—H1*N*4⋯O5^i^ and C17—H17⋯N3^ii^ hydrogen bonds, enclosing an 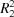
(8) ring motif and forming chains propagating along the *b*-axis direction (Fig. 2 ▸, Table 2 ▸). The chains are inter­connected *via* C17—H17*C*⋯O3^iii^ hydrogen bonds, forming a two-dimensional network parallel to the *ab* plane (Fig. 3 ▸). These chains are further stabilized by C12—H12*A*⋯*Cg*1^iii^ inter­actions, where *Cg*1 is the centroid of the Pt1/S1/N2/N3/C9 chelate ring (Fig. 3 ▸, Table 2 ▸). In addition, short inter­molecular O3⋯C7(−*x* + 3, −*y* + 1, −*z*) contacts of 2.897 (4) Å are observed; these are ∼0.32 Å shorter than the sum of van der Waals radii of carbon and oxygen atoms.

## Database survey   

A search of the Cambridge Structural Database (CSD version 5.40, last update May 2019; Groom *et al.*, 2016 ▸) using (*E*)-2-(2-(λ^1^-oxidan­yl)benzyl­idene)-*N*-cyclo­hexyl­hydrazine-1-carbo­thio­amide as the reference skeleton resulted in five related ligands containing cyclo­hexyl­hydrazine-1-carbo­thio­amide with different substituents. They include (*E*)-2-(***R*** **_1_**)-*N*-cyclo­hexyl­hydrazine-1-carbo­thio­amide, where ***R_1_*** = (2-hydro­naphthalen-1-yl)methyl­ene (BEFZIY; Basheer *et al.*, 2016 ▸), 5-bromo-2-hy­droxy-3-meth­oxy­benzyl­idene (LAQCIR; Jacob & Kurup, 2012 ▸), 4-(benz­yloxy)-2-hy­droxy­benzyl­idene (MOKPOT; Sajitha *et al.*, 2014 ▸), 5-chloro-2-hy­droxy­benzyl­idene (OBOLOJ; Arafath *et al.*, 2017*a* ▸), and 3-(*tert*-but­yl)-2-hy­droxy­benzyl­idene (YUXJOS; Arafath *et al.*, 2018 ▸). Selected geometrical parameters of **I** and the related structures are given in Table 3 ▸. As the ligand mol­ecule in **I** is chelated to platinum, it exists in a different tautomeric form. In **I**, the O1—C1 and S1—C9 bond lengths of 1.297 (3) and 1.745 (2) Å, respectively, are different from those in related ligands [O1—C1 and S1—C9 bond lengths in the ranges 1.350–1.362 and 1.683–1.693 Å, respectively]. As a chelating effect, the formation of the N3=C9 azomethine double bond is confirmed by its length [1.320 (3) Å], compared to 1.342–1.364 Å in related ligands. A decrease of the N2—N3—C9 angle is observed [119.03–121.82° compared to 113.79 (17)° in **I**]. Furthermore, the N2—N3—C9—N4 torsion angle in **I** has an *anti­periplanar* [−178.3 (2)°] conformation, whereas this torsion angle is in a *synperiplanar* [4.08–12.51°] conformation in the related ligands

## Synthesis and crystallization   

**Figure 4:**
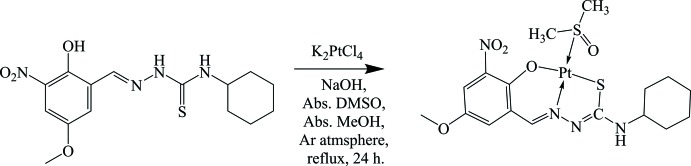
Reaction scheme for the synthesis of the title complex.

The reaction scheme for the synthesis of complex **I** is given in Fig. 4 ▸. The ligand (*E*)-*N*-cyclo­hexyl-2-(2-hy­droxy-5-meth­oxy-3-nitro­benzyl­idene)hydrazine-1-carbo­thio­amide (0.71 g, 2.00 mmol) was dissolved in 20 ml of methanol. A 2 mmol solution of NaOH in 10 ml of methanol was added and the mixture was refluxed for 30 min. A solution of K_2_PtCl_4_ (0.83 g, 2.00 mmol) was dissolved in 2 ml of DMSO and refluxed for 30 min. The resulting platinum(II) solution was added dropwise under stirring to the ligand solution under an Ar atmos­phere and refluxed for 24 h. The reddish-orange precipitate that formed was filtered off and washed with ethanol, ethyl acetate and *n*-hexane. It was then dissolved in chloro­form and aceto­nitrile (1:1) for recrystallization. Orange block-like crystals suitable for X-ray diffraction analysis were obtained on slow evaporation of the solvents (yield 88%, m.p. 510–511 K).

Analysis for C_17_H_24_N_4_O_5_PtS_2_ (FW: 623.61 g mol^−1^); calculated C, 32.71; H, 3.84; N, 8.97%; found: C, 32.67; H,3.76; N, 8.97%. IR (KBr pellets, cm^−1^): 3275 υ(NH), 3006 υ(CH_3_), 2927 and 2852 υ(CH, cyclo­hex­yl), 1582 υ(C=N), 1544 υ(C=C, aromatic), 1220 υ(C—S), 439 υ(Pt—N). ^1^H NMR (500 MHz, DMSO-*d*_6_, Me_4_Si ppm): δ 8.56 (*s*, HC=N), δ 7.60 (*d*, *J* = 7.55 Hz, CS—NH), δ 7.73 (*s*, H-aromatic), δ 7.70 (*s*, H-aromatic), δ 3.77 (*s*, Ph—OCH_3_), δ 2.54 [*s*, S(CH_3_)_2_], δ 1.92–1.12 (multiplet, N—C_6_H_11_). ^13^C NMR (125 MHz, DMSO-*d*_6_) δ 170.35 (C—S), δ 147.36 (C=N), δ 146.93–115.66 (C-aromatic), δ 56.14 (OCH_3_), δ 54.69 (N—C, cyclo­hex­yl), δ 40.42 [S(CH_3_)_2_], δ 32.44–24.75 (N—C_6_H_11_) ppm.

## Refinement   

Crystal data, data collection and structure refinement details are summarized in Table 4 ▸. The N-bound H atom was located in a difference-Fourier map and freely refined. The C*-*bound H atoms were positioned geometrically (C*—*H *=* 0*.*93–0.98 Å) and refined using a riding model with *U*_iso_(H) = 1.5*U*_eq_(C–meth­yl) and 1.2*U*_eq_(C) for other C*-*bound H atoms.

## Supplementary Material

Crystal structure: contains datablock(s) I, Global. DOI: 10.1107/S2056989019012623/su5513sup1.cif

Structure factors: contains datablock(s) I. DOI: 10.1107/S2056989019012623/su5513Isup2.hkl

CCDC reference: 1524712

Additional supporting information:  crystallographic information; 3D view; checkCIF report

## Figures and Tables

**Table 1:** Selected geometric parameters (Å, °)

Pt1—N2	1.9936 (17)	Pt1—S2	2.2254 (5)
Pt1—O1	2.0201 (15)	Pt1—S1	2.2441 (5)
			
N2—Pt1—O1	93.95 (6)	N2—Pt1—S1	84.74 (5)
N2—Pt1—S2	178.65 (5)	O1—Pt1—S1	178.63 (5)
O1—Pt1—S2	84.87 (5)	S2—Pt1—S1	96.45 (2)

**Table 2:** Hydrogen-bond geometry (Å, °) *Cg*1 is the centroid of chelate ring Pt1/S1/N2/N3/C9.

*D*—H⋯*A*	*D*—H	H⋯*A*	*D*⋯*A*	*D*—H⋯*A*
C17—H17*A*⋯O2	0.96	2.53	3.438 (4)	157
N4—H1*N*4⋯O5^i^	0.83 (3)	2.21 (3)	3.042 (2)	174 (2)
C17—H17*B*⋯N3^ii^	0.96	2.46	3.336 (3)	152
C17—H17*C*⋯O3^iii^	0.96	2.54	3.354 (4)	143
C12—H12*A*⋯*Cg*1^iii^	0.97	2.95	3.669 (3)	131

Symmetry codes: (i) 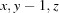; (ii) 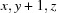; (iii) 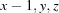.

**Table 3:** Selected geometrical parameters for **I** and related ligands (Å, °)

	**I**	BEFZIY	LAQCIR	MOKPOT*^*a*^*	OBOLOJ	YUXJOS
Pt1—O1	2.0201 (15)	–	–	–	–	–
Pt1—N2	1.9936 (17)	–	–	–	–	–
Pt1—S1	2.2441 (5)	–	–	–	–	–
Pt1—S2	2.2254 (5)	–	–	–	–	–
O1—C1	1.297 (3)	1.352	1.350	1.355, 1.350	1.360	1.362
S1—C9	1.745 (2)	1.693	1.685	1.683, 1.683	1.688	1.691
C6—C8	1.438 (3)	1.488	1.448	1.443, 1.448	1.459	1.457
C8—N2	1.300 (3)	1.294	1.268	1.281, 1.278	1.279	1.287
N2—N3	1.377 (2)	1.373	1.363	1.380, 1.379	1.369	1.388
N3—C9	1.320 (3)	1.354	1.342	1.346, 1.343	1.364	1.357
N4—C9	1.343 (3)	1.335	1.308	1.319, 1.323	1.325	1.336
N4—C10	1.460 (3)	1.467	1.455	1.464, 1.455	1.465	1.466
N2—N3—C9	113.79 (17)	121.82	120.90	121.70, 120.82	121.03	119.03
N3—C9—N4	116.41 (19)	117.02	116.60	117.35, 117.16	115.84	116.29
N2—N3—C9—N4	−178.3 (2)	−6.83	4.08	−6.04, 5.25	−5.50	12.51

Note: (*a*) MOKPOT crystallized with two independent mol­ecules in the asymmetric unit.

**Table 4:** Experimental details

Crystal data	
Chemical formula	[Pt(C_15_H_18_N_4_O_4_S)(C_2_H_6_OS)]
*M*_r_	623.61
Crystal system, space group	Triclinic, *P* 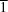
Temperature (K)	296
*a*, *b*, *c* (Å)	6.5264 (3), 8.9024 (3), 18.9261 (7)
α, β, γ (°)	82.228 (1), 87.074 (1), 74.739 (1)
*V* (Å^3^)	1050.96 (7)
*Z*	2
Radiation type	Mo *K*α
μ (mm^−1^)	6.91
Crystal size (mm)	0.49 × 0.21 × 0.15

Data collection	
Diffractometer	Bruker APEX Duo CCD area detector
Absorption correction	Multi-scan (*SADABS*; Bruker, 2012 ▸)
*T*_min_, *T*_max_	0.039, 0.092
No. of measured, independent and observed [*I* > 2σ(*I*)] reflections	41144, 6285, 5899
*R*_int_	0.030
(sin θ/λ)_max_ (Å^−1^)	0.711

Refinement	
*R*[*F* ^2^ > 2σ(*F* ^2^)], *wR*(*F* ^2^), *S*	0.019, 0.042, 1.09
No. of reflections	6285
No. of parameters	269
H-atom treatment	H atoms treated by a mixture of independent and constrained refinement
Δρ_max_, Δρ_min_ (e Å^−3^)	0.98, −0.80

Computer programs: *APEX2* and *SAINT* (Bruker, 2012 ▸), *SHELXS97* (Sheldrick, 2008 ▸), *Mercury* (Macrae *et al.*, 2008 ▸), *SHELXL2013* (Sheldrick, 2015 ▸) and *PLATON* (Spek, 2009 ▸).

## supplementary crystallographic information

### Crystal data

**Table:**

[Pt(C_15_H_18_N_4_O_4_S)(C_2_H_6_OS)]	*Z* = 2
*M**_r_* = 623.61	*F*(000) = 608
Triclinic, *P*1	*D*_x_ = 1.971 Mg m^−^^3^
*a* = 6.5264 (3) Å	Mo *K*α radiation, λ = 0.71073 Å
*b* = 8.9024 (3) Å	Cell parameters from 9889 reflections
*c* = 18.9261 (7) Å	θ = 2.4–30.2°
α = 82.228 (1)°	µ = 6.91 mm^−^^1^
β = 87.074 (1)°	*T* = 296 K
γ = 74.739 (1)°	Block, orange
*V* = 1050.96 (7) Å^3^	0.49 × 0.21 × 0.14 mm

### Data collection

**Table:**

Bruker APEX Duo CCD area detector diffractometer	6285 independent reflections
Radiation source: fine-focus sealed tube	5899 reflections with *I* > 2σ(*I*)
Graphite monochromator	*R*_int_ = 0.030
φ and ω scans	θ_max_ = 30.4°, θ_min_ = 2.2°
Absorption correction: multi-scan (SADABS; Bruker, 2012)	*h* = −9→9
*T*_min_ = 0.039, *T*_max_ = 0.092	*k* = −12→12
41144 measured reflections	*l* = −26→26

### Refinement

**Table:**

Refinement on *F*^2^	Primary atom site location: structure-invariant direct methods
Least-squares matrix: full	Secondary atom site location: difference Fourier map
*R*[*F*^2^ > 2σ(*F*^2^)] = 0.019	Hydrogen site location: mixed
*wR*(*F*^2^) = 0.042	H atoms treated by a mixture of independent and constrained refinement
*S* = 1.09	*w* = 1/[σ^2^(*F*_o_^2^) + (0.0179*P*)^2^ + 0.4664*P*] where *P* = (*F*_o_^2^ + 2*F*_c_^2^)/3
6285 reflections	(Δ/σ)_max_ = 0.001
269 parameters	Δρ_max_ = 0.98 e Å^−^^3^
0 restraints	Δρ_min_ = −0.80 e Å^−^^3^

### Special details

**Table:**

Experimental. The following wavelength and cell were deduced by SADABS from the direction cosines etc. They are given here for emergency use only: CELL 0.71080 6.582 8.984 19.105 82.236 87.079 74.752
Geometry. All esds (except the esd in the dihedral angle between two l.s. planes) are estimated using the full covariance matrix. The cell esds are taken into account individually in the estimation of esds in distances, angles and torsion angles; correlations between esds in cell parameters are only used when they are defined by crystal symmetry. An approximate (isotropic) treatment of cell esds is used for estimating esds involving l.s. planes.

### Fractional atomic coordinates and isotropic or equivalent isotropic displacement parameters (Å^2^)

**Table:**

	*x*	*y*	*z*	*U*_iso_*/*U*_eq_	
Pt1	0.43753 (2)	0.56002 (2)	0.24360 (2)	0.02894 (3)	
S1	0.16102 (9)	0.50827 (6)	0.30850 (3)	0.03473 (12)	
S2	0.37664 (9)	0.80884 (6)	0.26345 (3)	0.03468 (12)	
O1	0.6871 (3)	0.60194 (19)	0.18405 (10)	0.0390 (4)	
O2	0.9041 (4)	0.8000 (3)	0.14108 (15)	0.0723 (7)	
O3	1.1660 (4)	0.7358 (3)	0.07156 (15)	0.0768 (8)	
O4	1.2756 (3)	0.1801 (2)	0.04000 (12)	0.0554 (5)	
O5	0.1906 (3)	0.8775 (2)	0.30664 (12)	0.0536 (5)	
N1	1.0219 (3)	0.7032 (3)	0.10813 (12)	0.0431 (5)	
N2	0.4993 (3)	0.3368 (2)	0.22554 (9)	0.0269 (3)	
N3	0.3775 (3)	0.2401 (2)	0.25670 (11)	0.0328 (4)	
N4	0.0985 (3)	0.2204 (2)	0.33019 (12)	0.0383 (4)	
C1	0.8250 (3)	0.5008 (3)	0.15095 (12)	0.0313 (4)	
C2	0.9964 (4)	0.5447 (3)	0.11207 (12)	0.0343 (5)	
C3	1.1483 (4)	0.4417 (3)	0.07519 (13)	0.0394 (5)	
H3A	1.2590	0.4754	0.0510	0.047*	
C4	1.1352 (4)	0.2910 (3)	0.07449 (13)	0.0393 (5)	
C5	0.9686 (4)	0.2432 (3)	0.11050 (13)	0.0374 (5)	
H5A	0.9582	0.1415	0.1088	0.045*	
C6	0.8158 (3)	0.3422 (3)	0.14924 (12)	0.0315 (4)	
C7	1.4562 (4)	0.2214 (4)	0.00674 (16)	0.0520 (7)	
H7A	1.5477	0.1318	−0.0120	0.078*	
H7B	1.5316	0.2554	0.0411	0.078*	
H7C	1.4115	0.3049	−0.0314	0.078*	
C8	0.6590 (4)	0.2725 (3)	0.18624 (12)	0.0328 (4)	
H8A	0.6737	0.1675	0.1814	0.039*	
C9	0.2195 (3)	0.3102 (2)	0.29690 (12)	0.0299 (4)	
C10	−0.0798 (3)	0.2704 (3)	0.37886 (12)	0.0328 (4)	
H10A	−0.1680	0.3725	0.3583	0.039*	
C11	−0.2114 (4)	0.1513 (3)	0.38496 (14)	0.0409 (5)	
H11A	−0.2592	0.1448	0.3380	0.049*	
H11B	−0.1240	0.0486	0.4033	0.049*	
C12	−0.4028 (4)	0.1967 (4)	0.43416 (17)	0.0567 (8)	
H12A	−0.4989	0.2929	0.4127	0.068*	
H12B	−0.4778	0.1151	0.4396	0.068*	
C13	−0.3399 (5)	0.2203 (4)	0.50658 (17)	0.0617 (8)	
H13A	−0.2629	0.1201	0.5312	0.074*	
H13B	−0.4669	0.2597	0.5345	0.074*	
C14	−0.2027 (6)	0.3343 (4)	0.50108 (18)	0.0669 (9)	
H14A	−0.1554	0.3394	0.5482	0.080*	
H14B	−0.2864	0.4382	0.4827	0.080*	
C15	−0.0102 (4)	0.2858 (4)	0.45248 (15)	0.0479 (6)	
H15A	0.0806	0.1863	0.4730	0.058*	
H15B	0.0708	0.3637	0.4484	0.058*	
C16	0.6081 (5)	0.8331 (3)	0.30079 (16)	0.0496 (6)	
H16A	0.7288	0.7960	0.2707	0.074*	
H16B	0.6294	0.7741	0.3474	0.074*	
H16C	0.5912	0.9422	0.3044	0.074*	
C17	0.3707 (5)	0.9254 (3)	0.17999 (14)	0.0443 (6)	
H17A	0.5041	0.8925	0.1554	0.066*	
H17B	0.3461	1.0336	0.1871	0.066*	
H17C	0.2585	0.9137	0.1520	0.066*	
H1N4	0.133 (4)	0.127 (3)	0.3226 (15)	0.040 (7)*	

### Atomic displacement parameters (Å^2^)

**Table:**

	*U*^11^	*U*^22^	*U*^33^	*U*^12^	*U*^13^	*U*^23^
Pt1	0.03200 (4)	0.02243 (4)	0.03352 (5)	−0.00897 (3)	0.00631 (3)	−0.00618 (3)
S1	0.0360 (3)	0.0249 (2)	0.0439 (3)	−0.0090 (2)	0.0134 (2)	−0.0097 (2)
S2	0.0416 (3)	0.0244 (2)	0.0400 (3)	−0.0110 (2)	0.0086 (2)	−0.0094 (2)
O1	0.0397 (8)	0.0291 (8)	0.0510 (10)	−0.0137 (7)	0.0176 (7)	−0.0124 (7)
O2	0.0736 (15)	0.0459 (12)	0.107 (2)	−0.0319 (11)	0.0442 (14)	−0.0266 (12)
O3	0.0810 (16)	0.0737 (16)	0.0964 (19)	−0.0557 (14)	0.0474 (14)	−0.0303 (14)
O4	0.0482 (10)	0.0501 (11)	0.0665 (13)	−0.0118 (9)	0.0308 (9)	−0.0156 (10)
O5	0.0570 (11)	0.0342 (9)	0.0715 (14)	−0.0137 (8)	0.0292 (10)	−0.0213 (9)
N1	0.0440 (11)	0.0474 (12)	0.0446 (12)	−0.0246 (10)	0.0084 (9)	−0.0073 (10)
N2	0.0295 (8)	0.0234 (8)	0.0285 (9)	−0.0088 (6)	0.0058 (7)	−0.0040 (7)
N3	0.0364 (9)	0.0257 (8)	0.0395 (10)	−0.0137 (7)	0.0125 (8)	−0.0093 (7)
N4	0.0423 (10)	0.0287 (10)	0.0475 (12)	−0.0146 (8)	0.0196 (9)	−0.0136 (9)
C1	0.0318 (10)	0.0323 (11)	0.0307 (11)	−0.0099 (8)	0.0034 (8)	−0.0048 (8)
C2	0.0345 (10)	0.0372 (11)	0.0340 (11)	−0.0155 (9)	0.0036 (9)	−0.0039 (9)
C3	0.0338 (11)	0.0506 (14)	0.0356 (12)	−0.0151 (10)	0.0070 (9)	−0.0059 (10)
C4	0.0352 (11)	0.0435 (13)	0.0367 (12)	−0.0073 (10)	0.0092 (9)	−0.0060 (10)
C5	0.0378 (11)	0.0341 (11)	0.0391 (12)	−0.0086 (9)	0.0084 (9)	−0.0052 (9)
C6	0.0316 (10)	0.0319 (11)	0.0313 (11)	−0.0093 (8)	0.0063 (8)	−0.0055 (8)
C7	0.0377 (13)	0.0660 (18)	0.0514 (16)	−0.0123 (12)	0.0170 (11)	−0.0135 (14)
C8	0.0366 (11)	0.0263 (10)	0.0366 (12)	−0.0103 (8)	0.0092 (9)	−0.0077 (9)
C9	0.0307 (10)	0.0261 (10)	0.0349 (11)	−0.0099 (8)	0.0058 (8)	−0.0074 (8)
C10	0.0305 (10)	0.0301 (10)	0.0376 (12)	−0.0089 (8)	0.0093 (9)	−0.0056 (9)
C11	0.0385 (12)	0.0480 (14)	0.0409 (13)	−0.0214 (11)	0.0047 (10)	−0.0031 (11)
C12	0.0345 (12)	0.073 (2)	0.0617 (19)	−0.0205 (13)	0.0095 (12)	0.0031 (15)
C13	0.0555 (17)	0.074 (2)	0.0497 (17)	−0.0136 (15)	0.0238 (14)	−0.0007 (15)
C14	0.082 (2)	0.074 (2)	0.0501 (18)	−0.0261 (18)	0.0280 (16)	−0.0259 (16)
C15	0.0490 (14)	0.0564 (16)	0.0465 (15)	−0.0244 (13)	0.0084 (12)	−0.0166 (13)
C16	0.0622 (17)	0.0440 (14)	0.0489 (16)	−0.0207 (13)	−0.0070 (13)	−0.0120 (12)
C17	0.0620 (16)	0.0285 (11)	0.0433 (14)	−0.0135 (11)	−0.0024 (12)	−0.0033 (10)

### Geometric parameters (Å, º)

**Table:**

Pt1—N2	1.9936 (17)	C6—C8	1.438 (3)
Pt1—O1	2.0201 (15)	C7—H7A	0.9600
Pt1—S2	2.2254 (5)	C7—H7B	0.9600
Pt1—S1	2.2441 (5)	C7—H7C	0.9600
S1—C9	1.745 (2)	C8—H8A	0.9300
S2—O5	1.4694 (18)	C10—C11	1.521 (3)
S2—C17	1.765 (3)	C10—C15	1.522 (4)
S2—C16	1.774 (3)	C10—H10A	0.9800
O1—C1	1.297 (3)	C11—C12	1.519 (4)
O2—N1	1.212 (3)	C11—H11A	0.9700
O3—N1	1.213 (3)	C11—H11B	0.9700
O4—C4	1.370 (3)	C12—C13	1.507 (5)
O4—C7	1.418 (3)	C12—H12A	0.9700
N1—C2	1.456 (3)	C12—H12B	0.9700
N2—C8	1.300 (3)	C13—C14	1.511 (5)
N2—N3	1.377 (2)	C13—H13A	0.9700
N3—C9	1.320 (3)	C13—H13B	0.9700
N4—C9	1.343 (3)	C14—C15	1.521 (4)
N4—C10	1.460 (3)	C14—H14A	0.9700
N4—H1N4	0.83 (3)	C14—H14B	0.9700
C1—C2	1.419 (3)	C15—H15A	0.9700
C1—C6	1.434 (3)	C15—H15B	0.9700
C2—C3	1.391 (3)	C16—H16A	0.9600
C3—C4	1.369 (4)	C16—H16B	0.9600
C3—H3A	0.9300	C16—H16C	0.9600
C4—C5	1.385 (3)	C17—H17A	0.9600
C5—C6	1.395 (3)	C17—H17B	0.9600
C5—H5A	0.9300	C17—H17C	0.9600
			
N2—Pt1—O1	93.95 (6)	N3—C9—N4	116.41 (19)
N2—Pt1—S2	178.65 (5)	N3—C9—S1	124.17 (16)
O1—Pt1—S2	84.87 (5)	N4—C9—S1	119.42 (16)
N2—Pt1—S1	84.74 (5)	N4—C10—C11	107.99 (19)
O1—Pt1—S1	178.63 (5)	N4—C10—C15	113.0 (2)
S2—Pt1—S1	96.45 (2)	C11—C10—C15	109.9 (2)
C9—S1—Pt1	96.02 (7)	N4—C10—H10A	108.6
O5—S2—C17	109.52 (13)	C11—C10—H10A	108.6
O5—S2—C16	109.00 (14)	C15—C10—H10A	108.6
C17—S2—C16	101.25 (14)	C12—C11—C10	111.1 (2)
O5—S2—Pt1	119.46 (8)	C12—C11—H11A	109.4
C17—S2—Pt1	107.80 (9)	C10—C11—H11A	109.4
C16—S2—Pt1	108.28 (10)	C12—C11—H11B	109.4
C1—O1—Pt1	126.00 (14)	C10—C11—H11B	109.4
C4—O4—C7	117.3 (2)	H11A—C11—H11B	108.0
O2—N1—O3	120.7 (2)	C13—C12—C11	112.0 (2)
O2—N1—C2	121.1 (2)	C13—C12—H12A	109.2
O3—N1—C2	118.2 (2)	C11—C12—H12A	109.2
C8—N2—N3	116.09 (17)	C13—C12—H12B	109.2
C8—N2—Pt1	122.71 (14)	C11—C12—H12B	109.2
N3—N2—Pt1	121.16 (13)	H12A—C12—H12B	107.9
C9—N3—N2	113.79 (17)	C12—C13—C14	111.7 (3)
C9—N4—C10	126.23 (19)	C12—C13—H13A	109.3
C9—N4—H1N4	115 (2)	C14—C13—H13A	109.3
C10—N4—H1N4	118 (2)	C12—C13—H13B	109.3
O1—C1—C2	120.4 (2)	C14—C13—H13B	109.3
O1—C1—C6	124.07 (19)	H13A—C13—H13B	107.9
C2—C1—C6	115.6 (2)	C13—C14—C15	111.6 (3)
C3—C2—C1	123.0 (2)	C13—C14—H14A	109.3
C3—C2—N1	115.8 (2)	C15—C14—H14A	109.3
C1—C2—N1	121.2 (2)	C13—C14—H14B	109.3
C4—C3—C2	120.1 (2)	C15—C14—H14B	109.3
C4—C3—H3A	119.9	H14A—C14—H14B	108.0
C2—C3—H3A	119.9	C14—C15—C10	110.4 (2)
C3—C4—O4	125.0 (2)	C14—C15—H15A	109.6
C3—C4—C5	119.2 (2)	C10—C15—H15A	109.6
O4—C4—C5	115.8 (2)	C14—C15—H15B	109.6
C4—C5—C6	122.3 (2)	C10—C15—H15B	109.6
C4—C5—H5A	118.9	H15A—C15—H15B	108.1
C6—C5—H5A	118.9	S2—C16—H16A	109.5
C5—C6—C1	119.9 (2)	S2—C16—H16B	109.5
C5—C6—C8	115.4 (2)	H16A—C16—H16B	109.5
C1—C6—C8	124.71 (19)	S2—C16—H16C	109.5
O4—C7—H7A	109.5	H16A—C16—H16C	109.5
O4—C7—H7B	109.5	H16B—C16—H16C	109.5
H7A—C7—H7B	109.5	S2—C17—H17A	109.5
O4—C7—H7C	109.5	S2—C17—H17B	109.5
H7A—C7—H7C	109.5	H17A—C17—H17B	109.5
H7B—C7—H7C	109.5	S2—C17—H17C	109.5
N2—C8—C6	128.5 (2)	H17A—C17—H17C	109.5
N2—C8—H8A	115.8	H17B—C17—H17C	109.5
C6—C8—H8A	115.8		
			
C8—N2—N3—C9	179.0 (2)	C2—C1—C6—C5	−0.5 (3)
Pt1—N2—N3—C9	1.3 (3)	O1—C1—C6—C8	−2.8 (4)
Pt1—O1—C1—C2	−178.56 (16)	C2—C1—C6—C8	178.4 (2)
Pt1—O1—C1—C6	2.7 (3)	N3—N2—C8—C6	−177.9 (2)
O1—C1—C2—C3	−179.6 (2)	Pt1—N2—C8—C6	−0.2 (3)
C6—C1—C2—C3	−0.8 (3)	C5—C6—C8—N2	−179.5 (2)
O1—C1—C2—N1	−0.1 (3)	C1—C6—C8—N2	1.5 (4)
C6—C1—C2—N1	178.7 (2)	N2—N3—C9—N4	−178.3 (2)
O2—N1—C2—C3	−176.5 (3)	N2—N3—C9—S1	1.8 (3)
O3—N1—C2—C3	2.7 (4)	C10—N4—C9—N3	178.0 (2)
O2—N1—C2—C1	4.0 (4)	C10—N4—C9—S1	−2.2 (3)
O3—N1—C2—C1	−176.8 (3)	Pt1—S1—C9—N3	−3.4 (2)
C1—C2—C3—C4	0.9 (4)	Pt1—S1—C9—N4	176.77 (19)
N1—C2—C3—C4	−178.6 (2)	C9—N4—C10—C11	162.2 (2)
C2—C3—C4—O4	180.0 (2)	C9—N4—C10—C15	−76.0 (3)
C2—C3—C4—C5	0.4 (4)	N4—C10—C11—C12	−179.2 (2)
C7—O4—C4—C3	4.7 (4)	C15—C10—C11—C12	57.2 (3)
C7—O4—C4—C5	−175.7 (2)	C10—C11—C12—C13	−54.9 (3)
C3—C4—C5—C6	−1.7 (4)	C11—C12—C13—C14	53.0 (4)
O4—C4—C5—C6	178.7 (2)	C12—C13—C14—C15	−54.0 (4)
C4—C5—C6—C1	1.8 (4)	C13—C14—C15—C10	56.7 (4)
C4—C5—C6—C8	−177.2 (2)	N4—C10—C15—C14	−178.6 (2)
O1—C1—C6—C5	178.3 (2)	C11—C10—C15—C14	−57.9 (3)

### Hydrogen-bond geometry (Å, º)

*Cg*1 is the centroid of chelate ring Pt1/S1/N2/N3/C9.

**Table:**

*D*—H···*A*	*D*—H	H···*A*	*D*···*A*	*D*—H···*A*
C17—H17*A*···O2	0.96	2.53	3.438 (4)	157
N4—H1*N*4···O5^i^	0.83 (3)	2.21 (3)	3.042 (2)	174 (2)
C17—H17*B*···N3^ii^	0.96	2.46	3.336 (3)	152
C17—H17*C*···O3^iii^	0.96	2.54	3.354 (4)	143
C12—H12*A*···*Cg*1^iii^	0.97	2.95	3.669 (3)	131

Symmetry codes: (i) *x*, *y*−1, *z*; (ii) *x*, *y*+1, *z*; (iii) *x*−1, *y*, *z*.
